# Extensive long-term verbal memory training is associated with brain plasticity

**DOI:** 10.1038/s41598-021-89248-7

**Published:** 2021-05-06

**Authors:** Uttam Kumar, Anshita Singh, Prakash Paddakanya

**Affiliations:** 1 Centre of Bio-Medical Research, Sanjay Gandhi Postgraduate Institute of Medical Sciences Campus, Lucknow, Uttar Pradesh 226014 India; 2 Department of Psychology, Christ University, Bengaluru, India

**Keywords:** Neuroscience, Psychology

## Abstract

The human brain has a remarkable capacity to store a lifetime of information through visual or auditory routes. It excels and exceeds any artificial memory system in mixing and integrating multiple pieces of information encoded. In this study, a group of verbal memory experts was evaluated by multiple structural brain analysis methods to record the changes in the brain structure. The participants were professional Hindu pandits (priests/scholars) trained in reciting Vedas and other forms of Hindu scriptures. These professional Vedic priests are experts in memorization and recitation of oral texts with precise diction. Vedas are a collection of hymns. It is estimated that there are more than 20,000 mantras and shlokas in the four Vedas. The analysis included the measurement of the grey and white matter density, gyrification, and cortical thickness in a group of Vedic pandits and comparing these measures with a matched control group. The results revealed an increased grey matter (GM) and white matter (WM) in the midbrain, pons, thalamus, parahippocampus, and orbitofrontal regions in pandits. The whole-brain corelation analysis using length of post-training  teaching duration showed significant correlation with the left angular gyrus. We also found increased gyrification in the insula, supplementary motor area, medial frontal areas, and increased cortical thickness (CT) in the right temporal pole and caudate regions of the brain. These findings, collectively, provide unique information regarding the association between crucial memory regions in the brain and long-term practice of oral recitation of scriptures from memory with the proper diction that also involved controlled breathing.

## Introduction

Gaining new skills depends on the amount of practice and our propensity towards learning such skills. Multiple studies in human and animals have outlined that learning new skills is associated with structural brain plasticity in adults as well as during development^1–3^. However, the extent of structural changes with long-term training might build upon factors such as the amount of practice, the duration of practice, and the performance.

Several studies have shown that brain plasticity is associated with various kinds of skill training such as learning or practicing music, golf, video games, phonetics, driving, drumming, word learning, juggling, etc.^4–10^. These studies outlined a comprehensive outcome in terms of brain plasticity as a result of practicing specific skills. In the present study, we analyzed the brain plasticity associated with long-term practicing extensive memorization and verbal recitation of Vedic hymns, which is different from the skills mentioned above. For this purpose, we recruited Sanskrit Pandits/priests trained in Vedic schools for long years in oral recitation and memorization of Vedic hymns and associated subsidiary texts. Vedas are written in Sanskrit, an Indo-Aryan language. Pandits are primarily Hindu priests/scholars trained in Vedas and Vedic rituals. There are numerous *Vedshalas* (schools) in India imparting Vedic training in Sanskrit. It is a rigorous long-term course, mainly focusing on Vedas and religious texts. There are four branches of Veda: Rig-Veda, Yajur-Veda, Sama-Veda, and Atharva-Veda. Traditionally, Vedas have evolved through oral tradition and are believed to have 1180 recensions. Collectively, the four Vedas contain > 20,000 mantras and shlokas (approximately 50,000–100,000 words)^11^. Typically, the students attend *Vedshalas* from the age of seven or eight into their early twenties before graduating. The annual rate of dropout in such schools may be around 3–10%. The daily routine includes reciting, practicing and memorizing the various hymns for about 8–10 h. The recitation must follow the perfect diction, which requires a strong control over breath. The Vedas are also called *Srutis* because they are always received through hearing. While chanting the Veda, *swara* or accent is of great importance. If the accent changes, the meaning of the word might change totally, and hence, proper stress and diction are important. There are many methods of reciting Vedas such as *Pada Paatha*, wherein the sentence is broken down into words instead of stringing them together. *Krama Paatha,* which involves pairing the words successively and sequentially such as the first word to the second, the second to the third, the third to the fourth, and so on, and *Jata Paatha*, in which the first and the second words are first recited together and then recited in reverse order and then again in the original order^12^. This remarkable emphasis on recitation and memorization is extraordinary and unique to the form of education system followed in *Vedshalas*. The Veda learning skills integrate and facilitate multiple competencies such as excellent memory capacity, strong breath control, proper synchronization of motor articulator for accurate articulation, speech and hearing processing at phonemic and syllabic levels and coordination. We expected that the brain regions associated with the above functions show a plasticity effect.

A number of studies have highlighted that the hippocampus is a crucial region, which shows plasticity effects associated with memory^13–17^. Accumulating evidence affirm that the hippocampus also contributes to memory consolidation and spatial navigation by providing additional spatial coding and computations relevant for long-term memory^18,19^. However, these studies overlook the functional contribution of the other brain regions. The thalamus also has a long-standing link to memory. The subcortical circuit of the thalamus provides support to memory and spatial navigation^20,21^. The thalamic nuclei are also major components of the Papez circuit, as well as of the neural circuits responsible for specific categories of learning and memory^22,23^. The mammillothalamic tract lesion induce memory impairments, leading to amnesia^24–26^. Damages within this region are associated with Korsakoff syndrome, a chronic memory disorder^27,28^.

Pons and medulla oblongata are associated with the respiratory rhythm^29^, while the insular regions are linked with synchronization of speech articulators^30,31^. Previously, two studies have investigated the plasticity effects associated with extensive verbal memorization in Pandit samples. Kalamangalam and Ellomore^32^, analyzed the cortical thickness and reported changes in the left orbitofrontal and right inferior temporal region in a group of 11 Pandits/priests. Hartzell et al.^33^, reported multiple cortical and subcortical regions that showed a significant increase in GM, using whole-brain morphometry analysis. The study also reported that there was a marked increase in cortical thickness in multiple regions such as right superior temporal sulcus, right anterior temporal pole, right occipital-temporal gyrus, and left rostral anterior cingulate cortex, while the left inferior and middle occipital and right middle occipital gyrus showed reduced gyrification in the Pandit group. Differences in the findings between the studies could be due to the variation in selection method of participants, sample size, and methods adopted in the analysis. To further delve into the brain plasticity induced by verbal memorization and recitation practice, we studied a group of Pandits (N = 25) and an age-matched control group. We examined the data using voxel-based morphometry, cortical thickness, and local gyrification index.

Vedic teaching–learning takes place in a residential (gurukul) setup marked with a disciplined Hindu spiritualistic lifestyle and lasts for more than one and a half decades or more. Students enroll generally at a young age of 6 to 8 years of age. Pedagogically, the hallmark of Vedic schools is their emphasis on oral recitation and verbal memorization of Vedic hymns/texts and their interpretation. Such learning practices may impact one's verbal memory system and the underlying neural substrates that in turn may lead to a strong verbal memory system enhancing the overall efficiency to process even novel information. We planned this study to examine how the long-term aforesaid practice and skills impact the structural plasticity of the brain (malleability of the brain as a result of one's learning and experience). In the present study, all our Pandit participants belonged to the same ethnic group living in the same locality. Further, they were all from a single religion and caste. The pedagogical variation was controlled as all the Pandit participants were trained in the same Vedshala. The control group participants and Pandit participants were assessed on multiple behavioral and cognitive measures and matched on all the relevant variables. Most importantly, the Sanskrit proficiency of our comparison group participants was equal to that of Pandit participants. All these features of the study make it stronger as compared to such previously reported studies that had many confounding factors.

## Results

### Behavioral results

Among all the subtests of the PGI scale, we observed a significant difference only on the immediate recall. The performance of the Pandit/priest group (Mean: 11.16 ± 0.68) was significantly better than that of the control group (Mean: 8.72 ± 1.59; independent sample *t-*test (24) =  − 7.13, *p* < 0.00). The immediate recall score was found positively correlated with the GM volume of the thalamus (r = 0.51, *p* = 0.01). Though we performed the correlation analyses for all the behavioral scores with the GM values of the left thalamus, pons, and midbrain, we did not observe any significant correlations. The details are provided in the supplementary table (Table 1).

**Table 1:** Sample characteristics.

Characteristics	Pandit/priests	Control
Education history	Bachelor degree in Veda and Bachelor in education (Vedic)	Bachelor degree non-Veda/perusing master
Age (years)	24.95 (2.77)	23.13 (1.11)
Gender	Male	Male
Total year of learning	Average 11.13 years (Veda training) + 2 years post Veda training	Average 15 years
Teaching hour (weekly) (post-training)	21 h (Vedic teaching)	Not involve in teaching
Way of teaching	Verbal	
Language use in teaching	Hindi and Sanskrit	
Language known	Sanskrit, Hindi and English	Sanskrit, Hindi and English
Starting age of Vedshala	9 to 11 years (range)	
Starting age of training	10.58 (1.10)	
Total duration of Vedic training (h)	17,743.04 (4133.08)	
Ravens’ progressive matrix score	48.54 (3.78)	46.37 (3.59)^a^

^a^No significant differences exist.

### GM differences

We extracted the GM density of the regions identified in the VBM and whole brain correlation analyses by generating the region of interest (ROI) mask using WFU_PickAtlas. The regions included the left midbrain, pons, left thalamus, and left angular gyrus. We compared the GM value directly with that of the control group using independent sample “t” tests and found a significant difference between the groups in all the regions (t and *p* values for different regions: midbrain: t (24) = 2.60, *p* < 0.01; pons: t (24) = 2.90, *p* < 0.00; left thalamus: t (24) = 6.42, *p* < 0.00; and left angular gyrus: t (24) = 5.42, *p* < 0.00).

### VBM results

**Figure 1:**
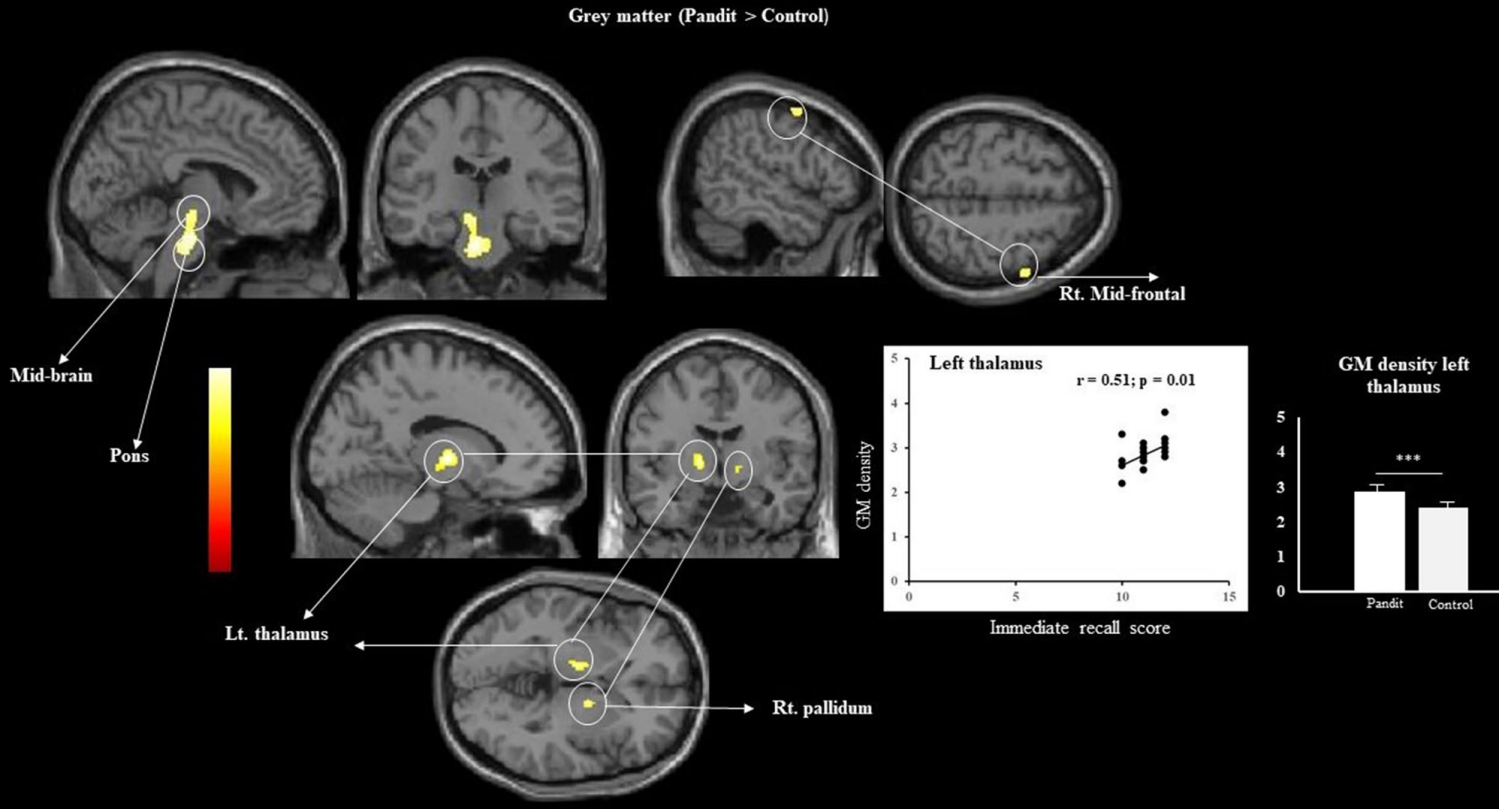
Results of VBM group comparison. The result rendered into standard stereotactic space and superimposed on to transvers, sagittal and coronal section of magnetic resonance image (MRI) which is itself in standard space. The image shows increased GM value in Pandit/Priest as a result of the two-sample *t*-test comparing with the Control, at a threshold of *P* < 0.05 (FWE) corrected. The figure also specifies GM density differences in two group and result of corelation analysis between GM density and immediate recall behaviour score.

**Figure 2:**
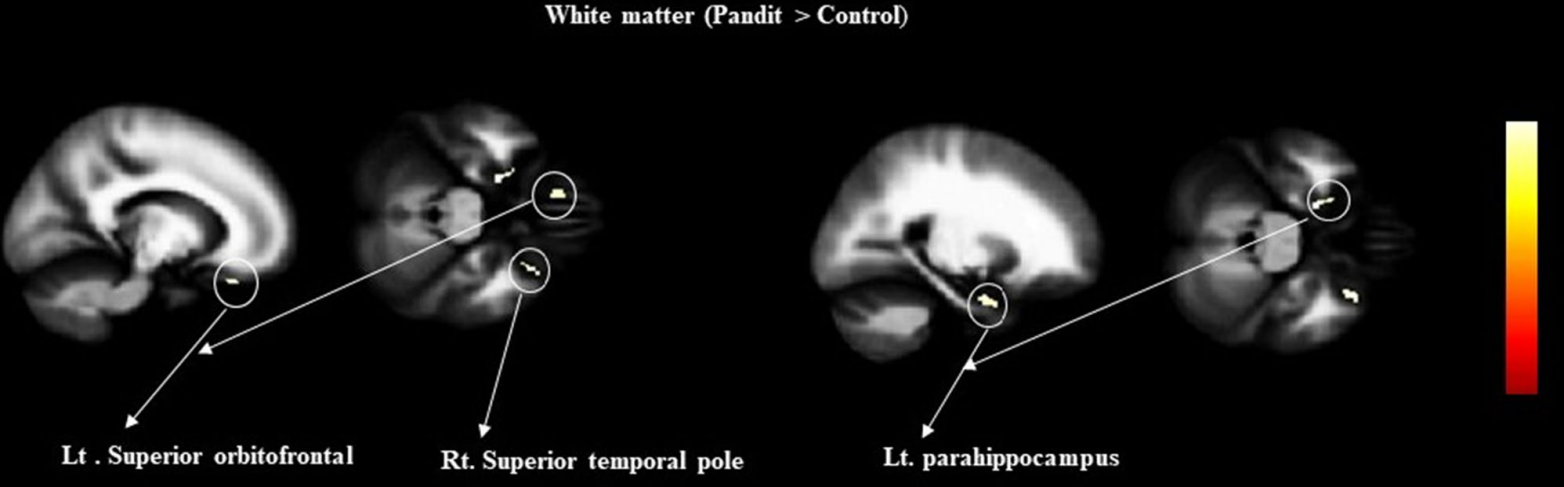
Results of VBM group comparison rendered into standard stereotactic space and superimposed on to magnetic resonance image (MRI) which is itself in standard space. The image shows increased WM value in Pandit/Priest as a result of the two-sample *t*-test comparing with the Control, at a threshold of *P* < 0.05 (FWE) corrected.

Figures 1, 2, and Tables 2 and 3 illustrate the VBM results of the direct comparison between the two groups. Analyses revealed an increased GM volume in the midbrain, pons, left thalamus, right ventral pallidum, and right frontal, while higher WM was located in the left parahippocampus, left superior orbitofrontal, and right superior temporal pole in Pandits. Controls did not show any increase in the GM and WM volume.

**Table 2:** Grey matter differences between Pandits/Priests and control based on VBM analysis (Pandit > control).

Anatomical regions	Hemisphere	T	Z	MNI coordinates		
				x	y	z
Mid brain	L	7.6	6.1	− 4	− 22	− 31
Pons	L	7.5	6.1	− 7	− 18	− 10
Thalamus	L	7.5	6.1	− 14	− 9	1.5
Pallidum	R	6.8	5.7	15	− 1	− 2
Middle frontal	R	6.0	5.2	54	1.5	57

**Table 3:** White matter differences between Pandits/priests and control based on VBM analysis (Pandit > control).

Anatomical regions	Hemisphere	T	Z	MNI coordinates		
				x	y	z
Parahippocampus	L	6.0	5.2	− 21	− 5	− 27
Temporal pole	R	5.8	5.0	31	16	− 28
Superior orbitofrontal	L	5.8	5.0	− 12	28	− 24

We also performed the whole brain corelation analyses taking into consideration the age of onset of Vedic training and length of post-training teaching duration (the approximate duration (in hours) of teaching as a Vedic teacher after completing the required Vedic training period). The onset age did not show any significant correlation with whole-brain analysis as no significant cluster survived. However, post-training duration showed significant correlation with the left angular gyrus GM voxel(MNI X, Y, Z coordinate: − 47, − 53, 35, Z value; 5.62) (*p* < 0.05/ FWE) (Fig. 5). We also extracted the GM density of the above region and performed the correlation analysis with post-training duration which showed a significant positive correlation (r = 0.43, *p* = 0.02).

### Cortical thickness and gyrification results

**Figure 3:**
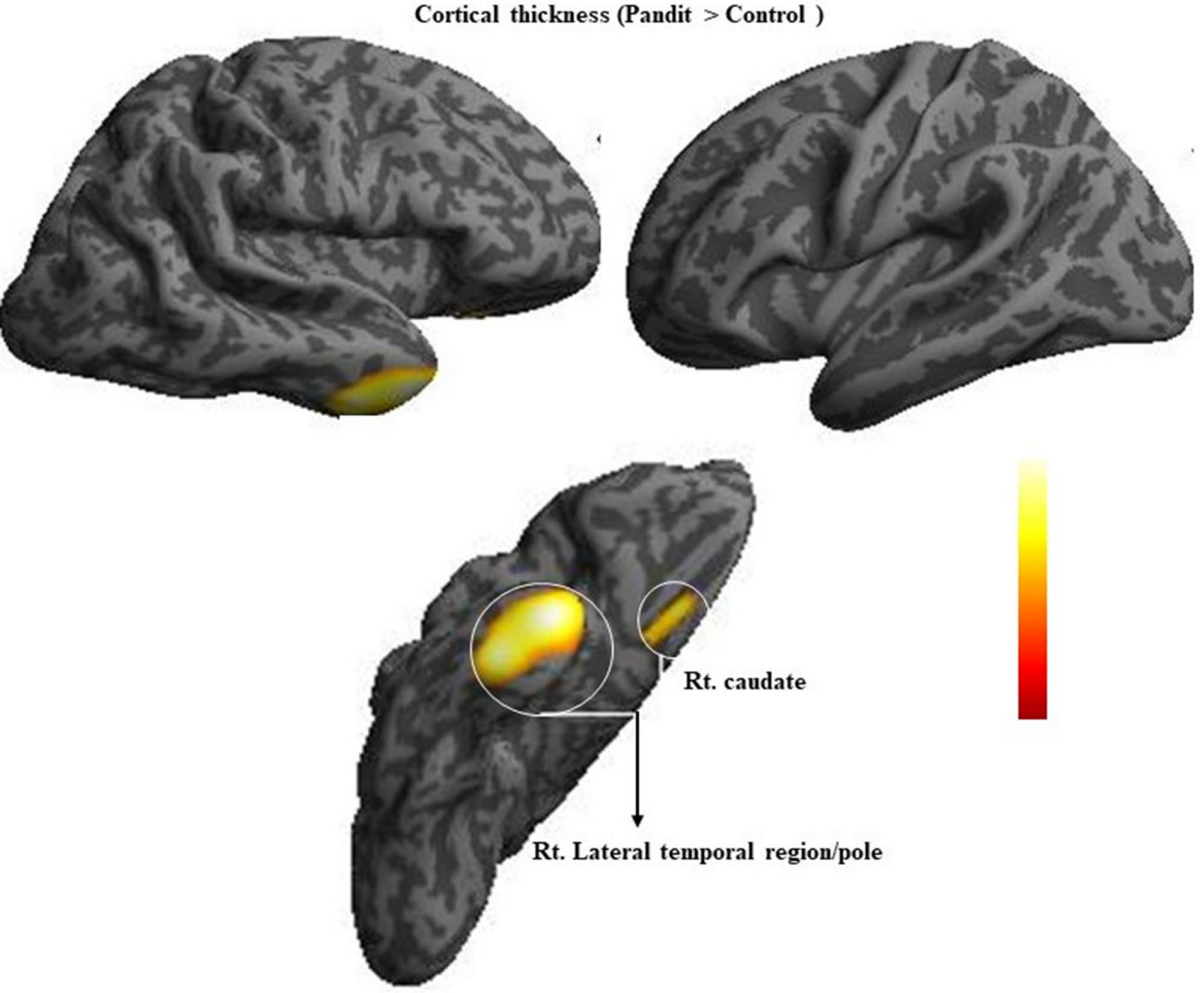
Results of projection based cortical thickness (PBCT) group comparison using computation anatomy toolbox (CAT) rendered on to an inflated cortical surface. The image shows increased cortical thickness in Pandit/Priest group based on two-sample *t*-test comparing with control. Thresholded at *P* < 0.05 (FWE) corrected.

**Figure 4:**
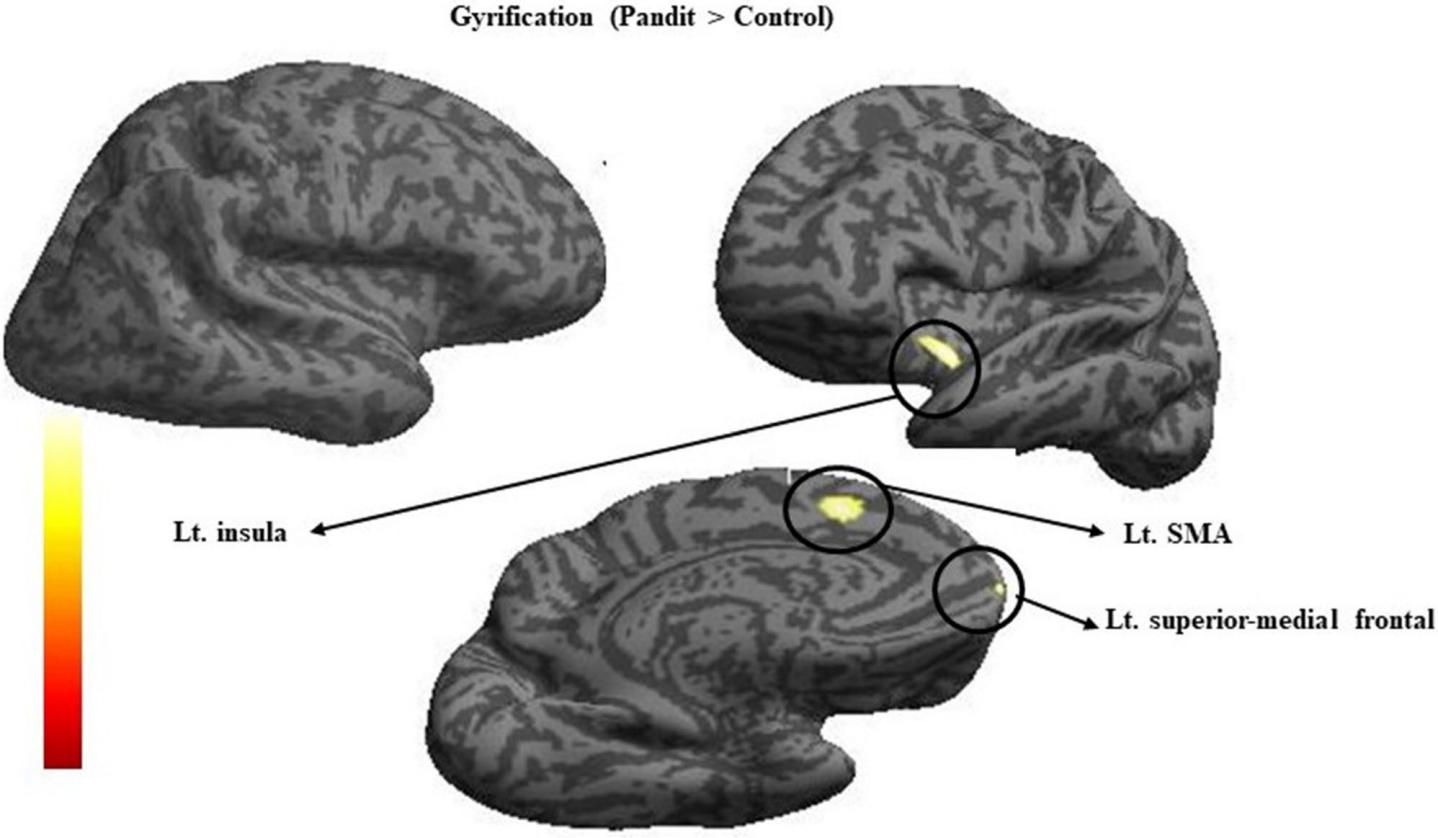
Results of gyrification group comparison using computation anatomy toolbox (CAT) rendered on to an inflated cortical surface. The image shows the result of the two-sample *t*-test comparing gyrification index value between Pandit/Priest, at a threshold of *P* < 0.05 (FWE) corrected.

Figure 3 and Table 4 show an increased cortical thickness in the right temporal pole and right caudate, while Fig. 4 and Table 5 show increased gyrification in the left insula, left supplementary motor area (SMA), and the left superior medial frontal region in the Pandit group.

**Table 4:** Brain regions with increased Cortical thickness in Pandits/Priests compared with Control (Pandit > control).

Anatomical regions	Hemisphere	T	Z	MNI coordinates		
				x	y	z
Temporal pole	R	8.2	6.1	41	9	− 15
Caudate	R	6.5	5.3	13	23	5

**Table 5:** Brain regions showing increased Cortical Gyrification in Pandits/Priests compared with control (Pandit > control).

Anatomical regions	Hemisphere	T	Z	MNI coordinates		
				x	y	z
Insula	L	5.6	4.7	− 34	4	9
Supplementary motor area	L	5.4	4.7	− 4	17	58
Superior medial frontal	L	4.7	4.1	− 5	57	29

**Figure 5:**
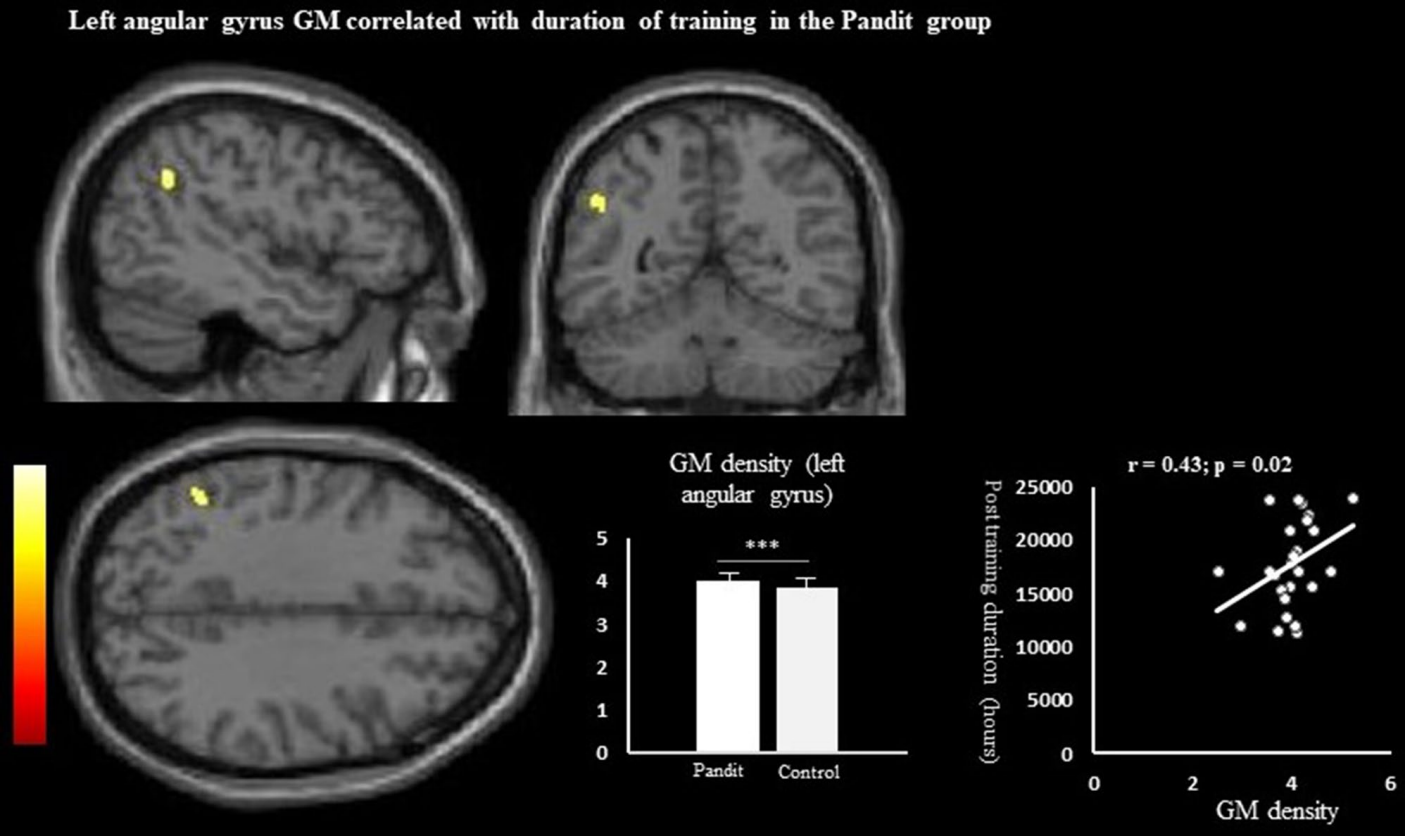
Result of the whole brain corelation analysis with GM value using length of post training teaching duration (hours) as a covariate in Pandit/Priest group. The result rendered into standard stereotactic space and superimposed on to transvers, sagittal and coronal section of magnetic resonance image (MRI) which is itself in standard space at a threshold of *P* < 0.05 (FWE) corrected. The figure also specifies GM density differences in two group.

We also examined the relationship between the LGI and CT findings. We created the mask regions based on the gyrification analysis and then calculated the group mean CT value and compared it between the groups. No significant group differences were observed between the groups. The mean CT values for Pandit group versus Control group were 2.63 mm (SD = 0.27) versus 2.74 mm (SD = 0.27); 2.87 mm (SD = 0.13) versus 2.83 mm (SD = 0.14); 2.73 (0.25) versus 2.62 (0.26) respectively for the above regions (Fig. 5).

## Discussion

In this study, we detected inter-group differences in the brain organization of professional Vedic Sanskrit Pandits/priests and a healthy control group using whole-brain comparative analysis. We observed a large GM cluster with increased volume in the midbrain, pons, left thalamus, right pallidum, and right frontal regions of the Pandit group. We found increased cortical thickness in the regions of right temporal pole and right caudate; and increased gyrification in the left insula, left SMA, and the left superior medial frontal area among Pandits. They also showed a higher WM volume in the left parahippocampus, left superior orbitofrontal, and right superior temporal pole. The behavior score revealed that Pandits showed a significantly better performance in immediate recall suggesting a superior ability with respect to conscious maintenance and use of information. Our findings are consistent with what we expected as all these regions have a direct link to the Vedic verbal recitation and memorization.

### GM alteration

A large number of earlier studies showed an association of the hippocampus with learning and memory. However, in this study, we did not observe any significant alteration in the GM of the hippocampus in Pandits; instead, we found GM alterations in the left thalamus. Thalamus is located in the diencephalon part of the forebrain and is a crucial part for the integration of sensory information as well as motor mechanism. The functional parcellation of the thalamus crumble due to technical pitfall^34,35^. However, the thalamic nuclei assist in major cortical function and higher-order cognition as it acts as a relay center subserving both motor and sensory mechanisms with a significant role in learning and memory. Any reorganization and plasticity effect in the thalamus are arbitrated by the corticofugal loop^36^. Studies affirm that thalamic maps are invariably acquainted with sensory experiences^37^. Learning and memory have a direct link and association with the thalamus because of the reciprocal connection with multiple regions as well as the Papez circuit^38^. The mediodorsal thalamus connects the medial temporal lobe and the orbitofrontal cortex, while the reuniens nucleus of thalamus is connected to the prefrontal cortex and temporal lobe^39,40^. Also, the thalamus is closely linked to the hippocampus because the anterior thalamic nuclei receive input directly from the hippocampal region^41^. This nucleus also receives input from mid-brain tegmentum which is crucial for learning and memory^42^. Several studies have established the significance of the mediodorsal thalamus in working memory because of its adjoining networking with the prefrontal cortex^43–45^. The dysfunction of thalamus impairs memory, and the condition is associated with Korsakoff syndrome^46^. The damaged thalamus also leads to the impairment in spatial memory tasks similar to the hippocampal damage^47^. The thalamic integrity is crucial for appropriate neural representation, and any damage to this region may also affect memory formation^48^. Another study demonstrated a significant contribution of the thalamus to monitoring, maintaining, and updating mental constructs^40^. This phenomenon highlights the dominant role of this region plays in memory and learning, and probably the rigorous memory exercise performed by the Pandits results in crucial GM alteration in the above region.

The other region, which showed high GM is the midbrain, bound rostrally by diencephalon to the midbrain accompanied by multiple small fiber tracts that relay useful information to the cerebral cortex, cerebellum, and brain stem. Thus, it plays a critical role in processing visual and auditory information along with motor control. The Vedic learning follows an oral tradition, which requires proper recitation with correct rhythm, syllabic foot, and measure of verse. The overt recitation or chanting involves auditory processing of heard sounds and then translating this auditory input into an articulatory output that exactly matches the heard sound. The entire process follows a close auditory integration. The inferior colliculus is the major auditory nucleus of the midbrain, and the excitatory and inhibitory synaptic input helps in shaping the neural response of sound in the midbrain^49^. Studies have affirmed a strong correlation between hippocampus and midbrain, specifically ventral tegmental and medial subtantia nigra, which plays a role in memory formation^49,50^. Together, it might induce GM alteration in the midbrain of the Pandits in the long-term.

Vedic mantra recitation is different from singing and talking as it follows a systematic method that requires a proper synchronization of stress, intonation, and breathing pattern. Breathing is a motor behavior and the lower brainstem i.e., pons and medulla are the regions that shape and adjust the breathing pattern^51–53^. This rigorous controlled breathing pattern during mantra recitation might increase the GM volume in the pons of the Pandits. Moreover, two other regions, middle frontal and ventral pallidum, were identified in the right hemisphere that showed increased GM volume. Reportedly, the middle frontal gyrus is involved in the reorientation of attention, attentional effort, and sustained attention^54–56^. Vedas are very complex with regard to their composition and lexical structures that required addional attentional effort. The chanting of Vedas is supposed to be related to motivation, reinforcement, and reward^57^, and the ventral pallidum that shows high GM is associated with reward and motivation^58,59^. The whole-brain GM correlation analysis with length of post-training teaching duration identified the left angular gyrus. Vedic teaching required that teachers recall and recite large number of Vedic Sanskrit hymns and verses accurately in the class as students had to learn reciting in the same manner by listening. Such teaching ability needed an exceptional memory retrieval skill and possibly this may be the reason that post training duration showed a positive correlation with GM value of angular gyrus. Earlier studies have indicated the crucial role of angular gyrus in memory retrieval using fMRI and other neuroimaging measures^60^ (For details about memory retrieval activity in angular gyrus see Rugg and King 2018)^61^.

### Cortical thickness

The cortical thickness offers an in-depth insight into the plasticity induced by learning new skills as it can be ascribed to the organization of cortical neurons. The thickness of the cortex varies from 2.3 to 2.8 mm in different cortical regions^62^. It provides information related to the density and arrangement of neurons, neuroglia, and nerve fibers^63^. In this study, we used the projection-based cortical thickness method^64^, and we found increased cortical thickness in the right temporal pole and right caudate in the Pandit group. The finding suggests that the temporal pole is closely related to the skills that are important in Vedic learning. The temporal pole canopies the anterior end of the temporal lobe and establishes a strong connection with the amygdala and orbital frontal cortex. The right temporal pole is specifically associated with emotions^65^ as damage to this region leads to socioemotional disorder^66^. The inherent meaning of Vedas and the information provided in them may have direct relevance to social-emotional learning^57,67,68^. Specifically, the Sam-Veda is considered as the Veda of melodies, chanting of which induces emotionally connected devotion^69^. However, there is no direct evidence to infer that the change in the temporal pole is related to a specific emotional measure. The other region with increased cortical thickness we found was the right caudate nucleus, a subcortical structure located deep inside the brain close to the thalamus^70^. A functional brain connectivity study suggested contralateral connectivity of right caudate with the thalamus specifically with the medial dorsal nucleus^71^. Another study reported that the right caudate is associated with the hippocampus in performance related to memory completion and working memory^72,73^. The increased thickness in the right caudate in the Pandit group might be due to these links.

### Gyrification

Gyrification analysis offers a novel approach to analyzing the brain as it targets morphometric properties, which are not captured by VBM or cortical thickness analyses. This technique is based on the GI that is the ratio of inner *vs*. outer cortical contours^74^. The analysis revealed three regions that show high GI in the left insula, left SMA, and left superior medial frontal in the Pandit group.

Interestingly, the style of recitation in Vedic learning is meant for better memorization and accurate pronunciation of words. Collectively, these methods use multiple phonetic rules, embedding special grammatical methods, and pairing words in sequences. The entire process follows correctly ordering the speech sounds at phonemic, syllabic, and supra-syllabic levels. These processes involve the cortical and subcortical speech regions. The insula and SMA are two regions that are frequently associated with motor aspects of speech production providing articulatory control. Insula establishes bilateral connectivity with linguistic, motor limbic, and sensory brain areas^75^. Some lesion-based studies affirmed that insula significantly supports motor articulation^76–78^. The contribution of SMA has been crucially involved in syllable sequence production^79,81^. Also, the SMA is a part of the network that contributes to the overt production of syllables^79^ and a crucial region of the sensorimotor circuits^82^. The association of these two regions with speech articulation might be the reason for observed increased gyrification in these regions. Numerous imaging studies have outlined the crucial role of the superior frontal gyrus in working memory^83–86^ and Vedic recitation, memorization, and ritual practice essentially require extensive use of working memory, which could be ascribed to high gyrification of this region.

### WM alteration

We measured the WM voxel value, which showed a marked increase in multiple regions, such as the left parahippocampus, left superior orbitofrontal, and right temporal pole in the Pandit group. The cerebral WM is a major contributing factor in bio-behaviors. VBM quantitively measures the WM volume, which is essential for normal systemic operations^87^. The Parahippocampus gyrus is a cortical region located in the medial temporal lobe that surrounds the hippocampus. Parahippocampus is closely connected to the hippocampus via the entorhinal cortex^88^. Some lesion-based studies on the human brain showed the important contribution to memory^89–91^, while the orbitofrontal cortex is strongly connected to the medial temporal cortex that supports in memory formation^92^, which might elevate the WM volume in Pandits/priests.

Vedic memorizing and recitation constitute multiple skills requiring a disciplined long-term commitment to master the Vedas. The coordination of such multiple skills requires the involvement of multiple neural regions. In the present study, we identified multiple regions that were implicated in the skills that are necessary for Vedic learning. Memorizing more than 50,000 to 100,000 words requires extraordinary learning skills, and this prompted us to identify multiple interconnected regions that are involved in the memory function.

The findings of the present study differ from the findings of the previous studies^32,33^ in certain respects. The previous VBM study^33^ located multiple regions that showed alteration in GM value, but none of those matches with the current study. The results also differ with respect to gyrification. Although CT analysis identified one common region i.e., temporal pole. The differences in findings between the studies may be due to multiple reasons but the main reason might be associated with the selection and matching of participants. In the present study, some naturalistic differences such as life-style between the groups could not be detangled. These social variabilities across the groups might have influenced biological signals of interest. Though we tried to match the groups on all relevant variables,.

## Conclusion

Multiple brain imaging studies have been carried out previously to highlight the importance of skill learning in the plasticity of the human brain's cortical and subcortical structures. This study provides insightful information regarding the functional consequences of acquiring new skills vis-a-vis neural regions. Three interrelated structural brain analysis methods such as VBM, cortical thickness, and gyrification index measurement suggest multiple regions that are associated with the plasticity effect of Vedic learning and recitation.

## Methods

### Participants

A total of 50 male volunteers (range 21–28 years) participated in the study conducted at the Centre of Biomedical Research, India. Of these, 25 professionally qualified Pandits were recruited from government-supported Vedic schools in the Lucknow (India) area. All the participants filled in the information regarding their extent of training, family history, current practice routines, lifestyle information, multilingualism, and handedness (Sample characteristic’s Table 1). The Pandits' qualifications included excellent dexterity in memorization and recitation ability. They all had begun their training at an early age (range 9–11 years**,** mean age 10.58 years) and were trained full-time for 10 years, for a total of approximately 17,743 h and continued training and reciting daily with reduced duration. They all are working as Vedic teachers and involve in contributing 21 h of weekly Vedic training. All the pandits rated themselves fluent in speaking, reading, and writing Sanskrit. Besides, 25 control volunteers matched on gender, age, Sanskrit proficiency, intellectual ability and the number of languages spoken, were recruited in this study. No minor participant (< 18 years old) participated in this study. All the participants were right-handed, as assessed using the Edinburgh Handedness Questionnaire^93^. Ravens’ progressive matrix was used to assess intellectual abilities of both the groups. The mean score of pandit group was 48.54 ± 3.78 and control group was 46.37 ± 3.59, albeit differences was not significant (t (24) =  − 1.88, *p* < 0.07). The study protocol was approved by the Centre of Biomedical Research Ethics Committee, and all participants provided written informed consent. All methods were performed in this study are in accordance with the relevant guidelines and regulations as recommended by the ethics committee.

Typically, the Pandits begin training at the government-supported Vedic schools between 6 and 12 years of age, without any requirement of an entrance examination. Daily Vedic training involves 06–07 h oral recitation/memorization practice, using the traditional face-to-face oral methods (*śruti-paramparā*). Upon successful learning, they graduate in 07 years with an additional 03 years of training in Vedic Sanskrit for them to become teachers.

### Behavioral assessment

Next, we conducted a PGI-Memory Scale (PGIMS) evaluation in all participants. This tool is standardized to the Indian population, and established a correlation of 0.71 with Boston Memory Scale and 0.85 with Wechsler Memory Scale. It assessed the following subsets: remote memory, recent memory, mental balance, attention and concentration, delayed recall, immediate recall, verbal retention for similar pairs, visual retention and recognition.

### MRI data acquisition

Data were collected on a 3 T Siemens Magnetom Skyra scanner at the Centre of Biomedical Research. A high-resolution three-dimensional T_1_-weighted image was collected using the following parameters: 192 slices; TR = 1600 ms; TE = 2.13 ms; flip angle = 12°; matrix = 224 × 256; 1 mm isotropic voxels. The image quality was reviewed to identify excessive movement, gradient non-linearity and B1 field inhomogeneity artifact.

### Statistical analysis

#### Voxel-based GM volume analysis

The structural data were processed and examined using the Statistical Parametric Mapping (SPM) voxel-based morphometry (VBM) 8 (Wellcome Department of Imaging Neuroscience Group, London, UK; http://www.fil.ion.ucl.ac.uk/spm) for VBM analyses in MATLAB 7.9. An optimized VBM^94^ protocol was applied for segmentation and normalization processes, using the DARTEL (Diffeomorphic Anatomical Registration using Exponentiated Lie algebra) toolbox for SPM^95^^.^ The SPM segmentation employed a mixture-model cluster analysis to identify voxel intensities matching specific tissue types. Subsequently, all the segmented GM and WM images were normalized to the GM and WM map and averaged using SPM mean function. A 14-mm full width at half-maximum (FWHM) isotropic Gaussian kernel was used to smoothen the images. Finally, voxel-wise groups were compared with respect to the smoothed GM and WM volumes using a two-sample *t*-test within SPM. The analysis was carried out using multiple comparison and familywise error correction (*FWE*). The *P*-value was set at < 0.05.

#### Projection-based cortical thickness (PBCT) and gyrification analysis

Cortical thickness and gyrification were estimated using an automated Computational Anatomy Toolbox (CAT12) (http://dbm.neuro.uni-jena.de/cat/). For cortical thickness, this method uses tissue segmentation to estimate the WM distance, and the distance from the inner boundary was estimated within the GM using a voxel-based distance method. This further provided a WM distance map, in which, the values at the outer GM boundary represent the GM thickness^64^. For gyrification analysis, local (vertex-wise) gyrification index (GI) was calculated based on the absolute mean curvature approach on the GM maps^96^. The mesh of the central surface was obtained by extraction of the cortical surface^97^, i.e., the surface between the GM/CSF border and the GM/WM boundary. The local absolute mean curvature of this central surface was calculated by averaging the mean curvature values from each vertex point. Moreover, the GI maps were smoothed with 15-mm FWHM. The comparison was carried out using multiple comparisons correction using *FWE* at *P* < 0.05.

## Supplementary Information

Supplementary Tables.
