# Supplementary material for: Extensive long-term verbal memory training is associated with brain plasticity
Source: Sci Rep. 2021 May 6;11:9712. doi: 10.1038/s41598-021-89248-7 (PMC8102627; doi:10.1038/s41598-021-89248-7)
Supplement: Supplementary file 1 — Supplementary Tables. [file 41598_2021_89248_MOESM1_ESM.docx]

**Extensive long-term verbal memory training is associated with brain plasticity**

Uttam Kumar ^1^, Anshita Singh^1^ & Prakash Paddakanya^2^

^1^Centre of Bio-Medical Research, Sanjay Gandhi Postgraduate Institute of Medical Sciences campus, Lucknow (India).

^2^Department of Psychology, CHRIST (Deemed to be University), Bengaluru (India)

| **Memory Scale** | **Mid Brain**  **Mean: 1.26 (0.18)** | **Pons**  **Mean:1.08 (0.22)** | **Thalamus (left)**  **Mean: 2.86 (0.31)** |
| --- | --- | --- | --- |
| Mental balance | r= .21  p= .30 | r= .31  p= .12 | r= .02  p= .89 |
| Attention concentration  (Forward) | r= -.23  p= .26 | r= -1.08  p= .60 | r= -.15  p= .45 |
| Attention concentration  (Backward) | r= .11  p= .59 | r= .16  p= .43 | r= .18  p= .38 |
| Delayed recall | r= .12  p= .54 | r= .37  p= .06 | r= .18  p= .36 |
| Verbal retention | r= -.06  p= .77 | r= -.10  p= .62 | r= -.30  p= .05 |
| Visual retention | r= .04  p= .83 | r= -.04  p= .82 | r= .12  p= .53 |
| Recognition | r= -.22  p= .27 | r= -.28  p= .16 | r= -.24  p= .24 |
| Immediate recall | r= -.044  p= .83 | r= -0.6  p= .77 | r= .51  p= .01 |

**Table 1: Result of PGI memory test correlation analyses with grey matter volume in pandit group.**

| **Scale** | **Pandit (25)** | **Healthy Control (25)** | **Significant**  **Differences** |
| --- | --- | --- | --- |
|  | **Mean (SD)** | **Mean (SD)** |  |
| Mental Balance | 6.95(1.41) | 5.75 (1.23) | NS |
| Attention and Concentration  Forward | 8.15(2.95) | 9.8(2.43) | NS |
| Attention and Concentration  Backward | 8.35(4.28) | 6.45(3.39) | NS |
| Delayed Recall | 7.55(0.87) | 6.85(1.43) | NS |
| Immediate Recall | 9(0.67) | 11.15(1.58) | .00 |
| Verbal Retention for Similar Pairs | 4.7(0.50) | 4.6(0.57) | NS |
| Visual Retention | 9.65(2.32) | 8.45(1.95) | NS |
| Recognition | 8.5(0.79) | 9(1.1) | NS |

**Table 2: PGI Memory Scale Assessments Score**
